# Crowded Out

**Published:** 1887-03

**Authors:** 


### CROWDED OUT.

The report of the January meeting of The Central Dental Association of Northern New Jersey is deferred till the appearance of our next number. Either both that and the report of the First District meeting must be divided, giving but a part of each in this number, or one of them must be held over. As the latter was first received, it is printed this month, and the other will appear in full in the April number.

